# Serum FT3/FT4, but not TSH is associated with handgrip strength in euthyroid U.S. population: evidence from NHANES

**DOI:** 10.3389/fendo.2024.1323026

**Published:** 2024-03-04

**Authors:** Zhizhuo Wang, Peiyun Wu, Jing Yang, Yuanshan Jiang, Juan Wang, Cheng Lin

**Affiliations:** Department of Rehabilitation Medicine, School of Health, Fujian Medical University, Fuzhou, China

**Keywords:** FT3/FT4 ratio, thyroid-stimulating hormone, handgrip strength, euthyroid, NHANES

## Abstract

**Objective:**

Although several studies have examined the relationship between thyroid function and muscle strength, their population primarily derived from Asian areas, and their results were controversial. Thus, this study aimed to explore the association between thyroid function and handgrip strength (HGS) in the U.S. population.

**Methods:**

A total of 1,067 participants from NHANES were categorized into three different age groups including young (<45 years), middle (45~64 years), and old (≥65 years) age groups. Thyroid function was measured by the competitive binding immune-enzymatic assays, while HGS was examined by a trained evaluator using a dynamometer. The weighted multiple linear regression models were used to examine the association between thyroid function and handgrip strength. The restricted cubic splines were employed to explore the non-linear relationship between these two variables. All statistical analyses were performed using the SPSS version 20.0 and R software.

**Results:**

After adjustment for potential covariates, FT3/FT4, but not TSH was positively associated with HGS in middle age group (β=0.091, t=2.428, P=0.016). The subgroup analysis by sex revealed that the positive association between FT3/FT4 and HGS was observed in the middle age group for both male and female participants (β=0.163, t=2.121, P=0.035; β=0.157, t=2.180, P=0.031). The RCS analysis showed a statistically significant non-linear association between FT3/FT4 and HGS in overall population (P for non-linear=0.026). After adjustment for covariates, men with low HGS had a significant lower FT3/FT4 than those without low HGS in old age group (P=0.013). There was a significant increase in TSH level for female participants with low HGS in old age group compared to those with normal HGS (P=0.048).

**Conclusions:**

This study demonstrated FT3/FT4, but not TSH, was positively associated with HGS in middle age group, and the different association was observed in men in middle age group when participants were stratified by sex. Future longitudinal cohort study should be conducted to reveal the causal relationship between thyroid function and muscle strength.

## 1 Introduction

The butterfly-shaped thyroid gland secrets thyroid hormones, primarily including triiodothyronine (T3) and thyroxine (T4), which contributes to maintaining the normal healthy development of the brain, heart, and other essential organs by controlling body’s metabolism, regulating lipid profiles, and maintaining the normal reproductive function, etc. (1). Thyroid-stimulating hormone (TSH), synthesized by adenohypophysis, modulates the biosynthesis and secretion of T3 and T4 in the thyroid. In turn, TSH secretion is regulated by negative feedback action of T3 and T4. There are two forms of T3 and T4 in peripheral blood: free and binding forms. The free T3 (FT3) and T4 (FT4) interact with target cells to exert their biological functions. Furthermore, the conversion of T4 to T3 is catalyzed by Type 1 (D1) and 2 (D2) iodothyronine deiodinases (2). Abnormal non-pathological thyroid hormone variation may lead to serious diseases. A growing body of evidence has shown that hypothyroidism is correlated with hypertension, cardiovascular disease, and neurocognitive deficits (3–5), while hyperthyroidism is associated with atrial fibrillation and coronary vasospasm (6, 7).

Muscle strength is an essential health-related factor of fitness, and the decline in muscle strength is related to higher mortality and morbidity (8, 9). Handgrip strength (HGS) is a simple, reliable, and favorable measurement of the overall muscle strength with a hand squeezing around a dynamometer (10). Skeletal muscle functions as one of main targets for thyroid hormones, which impacts body composition and physical performance through T3 receptor of mitochondria (11). Recent studies have shown that the condition of muscle strength might correlate with thyroid function. A cross-sectional study of the sixth KNHANES (Korean National Health and Nutrition Examination Survey) revealed that serum TSH level was significantly associated with lower HGS in men, suggesting that the changes in thyroid function had a distinct impact on muscle in Korean men adults (12). In addition, a recent study of the TCLSIH (Tianjin Chronic Low-grade Systemic Inflammation and Health) confirmed that high-normal FT3 and higher FT3/FT4 ratios significantly associated with annual change in HGS among middle-aged and older euthyroid adults (13). Although several studies have examined the relationship between thyroid function and muscle strength, their population primarily derived from Asian areas, and their results were controversial. Thus, this study aimed to explore the association between thyroid function and HGS through retrieving data from the National Health and Nutrition Examination Survey (NHANES) where the sample represents the civilian noninstitutionalized U.S. population. It is hypothesized that thyroid hormone profile might influence muscle strength. This study might render new insights on muscular fitness management from a homeostatic perspective.

## 2 Materials and methods

### 2.1 Study population and design

The National Health and Nutrition Examination Survey (NHANES) is a nationwide and ongoing cross-sectional survey administrated by the U.S. National Center for Health Statistics. The repeated two-year cycle test was conducted by employing a complex multistage probability sampling design among the civilian noninstitutionalized U.S. population. The NHANES was approved by the National Center for Health Statistics Research Ethics Review Board and all written informed consent had been received from recruited participants.

**Figure 1:**
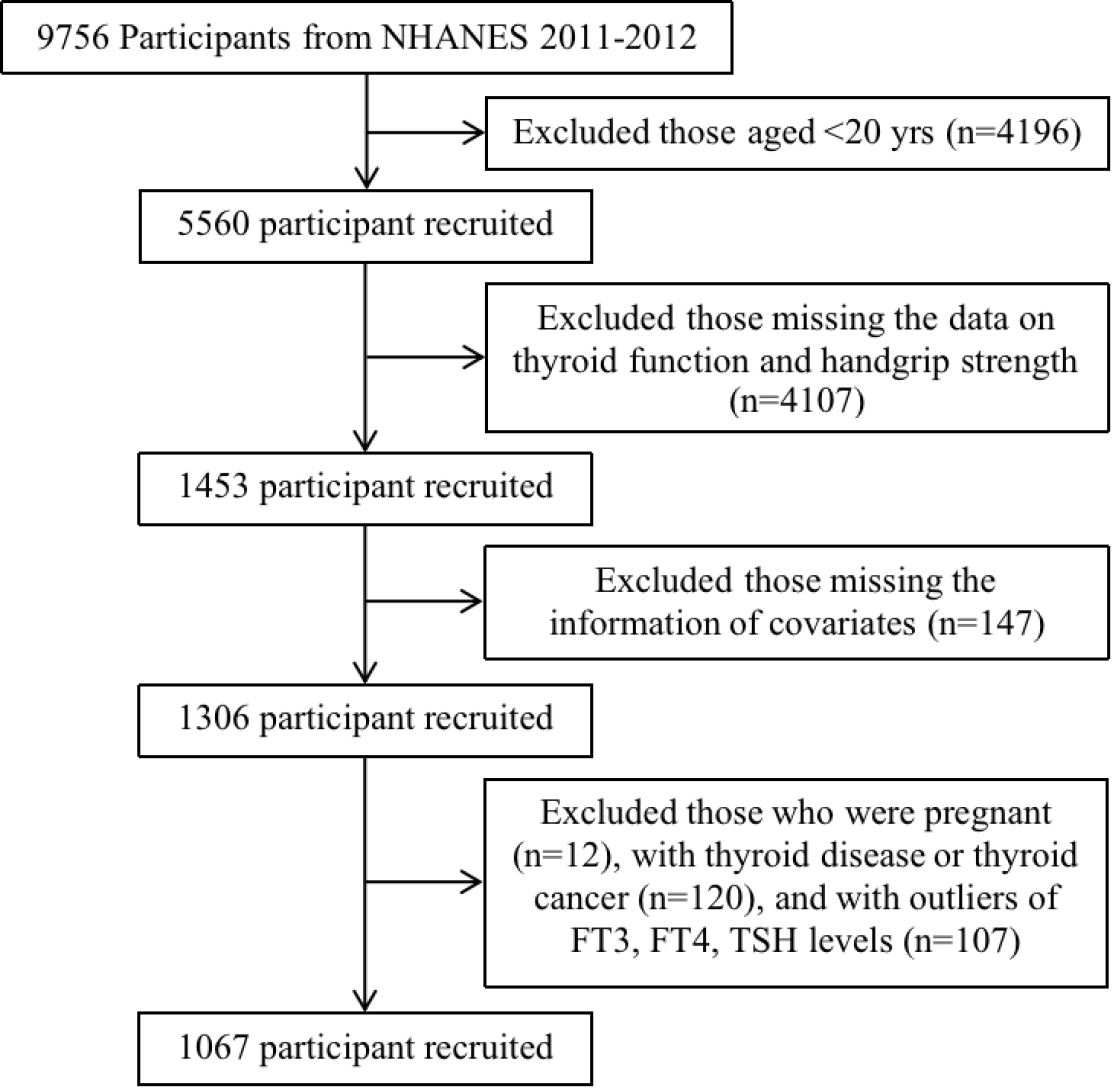
The flowchart of participants’ inclusion and exclusion process.

The data of this study were retrieved from NHANES 2011-2012 survey cycle due to the fact that only this cycle involved the complete information about thyroid function and handgrip strength. In total, there were 9,756 participants in the NHANES 2011-2012. We excluded participants who were aged less than 20 years (n=4,196), without the data of investigated independent variables (i.e., thyroid function and handgrip strength) (n=4,107), with the missing information of covariates (sex, race, educational level, marital status, smoking status, drinking status, blood pressure, urine iodine concentration, body mass index) (n=147). Besides, women who were pregnant (n=12), participants with thyroid disease or thyroid cancer (n=120), and participants with outliers of FT3, FT4, TSH levels (n=107) were excluded. Ultimately, a total of 1,067 participants were included in our study (**Figure 1**). The included participants were categorized into three different age groups including young (<45 years), middle (45~64 years), and old (≥65 years) age groups.

### 2.2 Thyroid function

Thyroid function data in the study were obtained from NHANES laboratory section, including FT3, FT4 and TSH. A competitive binding immune-enzymatic assay was employed for measuring FT3 and FT4. The measurement of TSH was performed using a third generation two-site immune-enzymatic (“sandwich”) assay. The reference range of FT3, FT4 and TSH was 3.84~5.99 pmol/L, 7.72~20.60 pmol/L, 0.34~5.60 mIU/L, respectively (14). Detailed description of laboratory methodology was seen on the website (https://wwwn.cdc.gov/Nchs/Nhanes/2011-2012/THYROD_G.htm).

### 2.3 Handgrip strength

Handgrip strength (HGS) was examined by a trained evaluator using a dynamometer (Takei Digital 5401; Takei Scientific Instruments, Tokyo, Japan). The participants were instructed to be positioned sitting upright with their arms hanging on the body side and squeeze the dynamometer using the dominant hand and non-dominant hand for strength testing. Three repeated measurements were implemented with an interval of 60 seconds between each measurement, and the highest grip-strength value was recorded for further analysis. Handgrip strength was expressed in kilograms (kg) and calculated as the maximum value out of three handgrip testing attempts in the dominant hand. The individual-based reference value for dominant HGS was based on the following equations proposed by Wang et al. (15): for male, grip strength = −29.959 – 3.095 × 10^−5^ × (Age^3^) + 38.719 × (Height) + 0.113 × (Weight); for female, grip strength = –22.717 – 1.920 × 10^−5^ × (Age^3^) + 30.360 × (Height) + 0.048 × (Weight). As such, we compared the measured maximum HGS of the dominant hand with the reference value. If the maximum HGS was lower than the reference value, participants were defined as low HGS, otherwise as normal HGS.

### 2.4 Covariates

The covariates incorporated in the study were as follows: sex (male, female), race/ethnicity (Mexican American, Other Hispanic, Non-Hispanic White, Non-Hispanic Black, Other Race), educational level (below high school, high school, above high school), marital status (married/living with partner, widowed/divorced/separated/never married), drinking status (yes, no), smoking status (never, former, current), hypertension (yes, no), urine iodine concentration (UIC, categorized into <100ug/L as iodine deficient, 100~299ug/L as normal, ≥300ug/L as excessive iodine intake), and body mass index (BMI, grouped into <25kg/m^2^ as normal, 25~29.9kg/m^2^ as overweight, ≥30kg/m^2^ as obese). The triple classifications of smoking status were based on the “Smoking–Cigarette Use” questionnaire where participants are asked if they have smoked at least 100 cigarettes in their entire lives. If the question was answered “no”, they were classified as a never smoker, otherwise they were followed with another question (“Do you now smoke cigarettes?”) to be further subclassified. The answer “no” or “yes” of the followed question determined whether someone was classified as a former smoker or a current smoker, respectively (16). Participants were diagnosed with hypertension if they satisfied one of the following criteria (17): 1) the question (“ever told you had high blood pressure, also called hypertension?”) was answered “yes”; 2) the question (“taking prescription for hypertension?”) was responded “yes”; 3) the mean reading of three consecutive blood pressure measurements reached the threshold of defining hypertension (systolic blood pressure ≥140 mmHg and/or diastolic blood pressure ≥90 mmHg). BMI was calculated as body weight (kg) divided by the square of height (m^2^), and the categorization referred to the previous published literature (18). UIC was divided into three different groups according to iodine concentration of participants, which could impact the thyroid function (19).

### 2.5 Statistical analysis

Survey sampling weights were considered for the complex multistage probability sampling design to generate nationally representative estimates (20). For the baseline characteristics of participants, continuous variables were expressed as weighted mean ± standard deviation (SD), while categorical variables were presented as counts (weighted percentages) in this study. the weighted one-way ANOVA test (for continuous data) and weighted Chi-square test (for categorical data) were employed to compare differences among different age groups. Weighted multivariable linear regression models were applied to test the associations between thyroid function including two indices (FT3/FT4 and TSH) and HGS. Model 1 was crude adjusted (unadjusted); Model 2 was adjusted for sex, race/ethnicity, educational level, marital status; Model 3 was further adjusted for smoking status, drinking status, hypertension, BMI, UIC. Considering the impact of sex on thyroid function (21), the subgroup analyses by sex categories were performed with all covariates except itself adjusted. A restricted cubic spline regression (RCS) was used to explore the non-linear relationship between thyroid function and HGS. All statistical analyses were performed using the SPSS version 20.0 (SPSS Inc., Chicago, IL, USA), and R software (version 4.1.0, R Foundation for Statistical Computing). P<0.05 was considered as statistical significance.

## 3 Results

### 3.1 Baseline characteristics of participants

The characteristics of participants by different age groups were shown in **Table 1**. Of the 1,067 included participants, the young age group (<45 years) contained 489 participants, the middle age group (45~64 years) involved 371 participants, and the old age group (≥65 years) constituted 207 participants, whereas the average of age in each group was 31.94 (SD=7.60), 53.92 (SD=5.61), and 72.60 (SD=5.06), respectively. Among the three different age groups, for sociodemographic information, the differences in educational level and drinking status were statistically significant (P<0.001). In term of examination and laboratory data, the differences with statistical significance (P< 0.01) were hypertension, HGS, UIC, FT3, FT4, and TSH. Moreover, the average of FT3/FT4 was 0.46 ± 0.07, and participants in old age group tended to have a lower FT3/FT4 level (0.43 ± 0.67) compared with young and middle age groups (P<0.001). However, an increasing tendency was shown for the average of TSH level from young to old age group (P for trend <0.001).

**Table 1:** Characteristics of study participants by different age groups.

Variables	Young age	Middle age	Old age	P*-*value
Number of participants	489	371	207	
Age (yrs), mean ± SD	31.94 ± 7.60	53.92 ± 5.61	72.60 ± 5.06	<0.001
Sex, %				
Male	283 (55.60)	192 (49.56)	116 (54.49)	0.198
Female	206 (44.40)	179 (50.44)	91 (45.51)	
Race/Ethnicity, %				
Mexican American	56 (12.00)	39 (5.57)	8 (1.79)	0.614
Other Hispanic	48 (9.26)	47 (6.45)	25 (5.36)	
Non-Hispanic White	169 (57.93)	122 (70.38)	95 (80.73)	
Non-Hispanic Black	114 (11.85)	109 (11.80)	52 (7.54)	
Other Race	102 (8.96)	54 (5.80)	27 (4.58)	
Educational level, %				
Below high school	75 (12.84)	84 (13.56)	84 (30.45)	<0.001
High school	87 (17.97)	81 (19.43)	34 (16.88)	
Above high school	327 (69.19)	206 (67.01)	89 (52.67)	
Marital status, %				
Married/Living with partner	261 (55.99)	220 (65.11)	117 (63.74)	0.220
Widowed/Divorced/Separated/Never married	228 (44.01)	151 (34.89)	90 (36.26)	
Smoking status, %				
Never	332 (66.05)	198 (55.01)	87 (38.38)	0.817
Former	66 (16.96)	89 (21.05)	89 (48.78)	
Current	91 (16.99)	84 (23.94)	31 (12.84)	
Drinking status, %				
Yes	398 (85.53)	275 (81.95)	139 (73.39)	<0.001
No	91 (14.47)	96 (18.05)	68 (26.61)	
BMI, %				
<25kg/m^2^ (Normal)	176 (35.25)	85 (21.29)	69 (35.31)	0.090
25~29.9kg/m^2^ (Overweight)	153 (34.25)	128 (35.74)	73 (34.42)	
≥30kg/m^2^ (Obese)	160 (30.50)	158 (42.97)	65 (30.27)	
Hypertension, %				
Yes	82 (15.00)	191 (46.80)	154 (68.91)	<0.001
No	407 (85.00)	180 (53.20)	53 (31.09)	
UIC, %				
<100ug/L (Iodine deficient)	202 (40.56)	140 (37.34)	52 (23.19)	0.002
100~299ug/L (Normal)	212 (44.22)	177 (50.41)	118 (58.61)	
≥300ug/L (Excessive iodine intake)	75 (15.22)	54 (12.25)	37 (18.20)	
Thyroid function				
FT3 (pmol/L), mean ± SD	5.04 ± 0.44	4.75 ± 0.43	4.58 ± 0.39	<0.001
FT4 (pmol/L), mean ± SD	10.72 ± 1.56	10.50 ± 1.49	10.97 ± 1.52	<0.001
FT3/FT4, mean ± SD	0.48 ± 0.07	0.46 ± 0.07	0.43 ± 0.67	<0.001
TSH (mIU/L), mean ± SD	1.62 ± 0.84	1.74 ± 0.86	2.01 ± 0.93	<0.001
HGS (kg), mean ± SD	41.38 ± 11.73	36.10 ± 10.59	30.97 ± 9.17	<0.001

Continuous variables were presented as weighted mean ± SD (Standard deviation), and categorical variables were presented as counts (weighted percentage), unless otherwise indicated; NHANES, National Health and Nutrition Examination Survey; HGS, Handgrip strength; BMI, Body mass index; UIC, Urine iodine concentration; FT3, Free triiodothyronine; FT4, Free thyroxine; TSH, Thyroid-stimulating hormone.

### 3.2 Association between thyroid function and HGS among different age groups

As shown in **Table 2**, weighted multivariable linear regression was employed to examine the association between thyroid function and HGS for participants by different age groups. In the unadjusted model, FT3/FT4 was positively associated with HGS in young and middle age groups (β=0.126, t=2.884, P=0.004; β=0.221, t=4.309, P<0.001). Importantly, the positive relationship between TSH and HGS was merely revealed in middle age group (β=0.129, t=2.397, P=0.017). After adjusting covariates of sex, race/ethnicity, educational level, marital status in Model 2, we found that a statistically significant association remained positively for FT3/FT4 in middle age group (β=0.095, t=2.505, P=0.013). Furthermore, this association remained remarkable between FT3/FT4 and HGS in middle age group when we employed a fully adjusted model (Model 3) (β=0.091, t=2.428, P=0.016), while the association for TSH with HGS was no longer present in either Model 2 or Model 3. However, no statistically significant association was found between FT3/FT4 and HGS in old age group (P>0.05).

**Table 2:** Association between thyroid function and HGS according to different age groups.

		Young age	Middle age	Old age
		Model 1		
FT3/FT4 with HGS	Standardized β	0.126	0.221	-0.015
	Standard Error	7.366	7.874	9.448
	*t* Value	2.884	4.309	-0.213
	*p* Value	**0.004**	**<0.001**	0.832
TSH with HGS	Standardized β	-0.035	0.129	-0.275
	Standard Error	0.631	0.636	0.690
	*t* Value	-0.765	2.397	-0.275
	*p* Value	0.445	**0.017**	0.738
		Model 2		
FT3/FT4 with HGS	Standardized β	0.012	0.095	0.071
	Standard Error	4.462	5.824	6.735
	*t* Value	0.440	2.505	1.371
	*p* Value	0.660	**0.013**	0.172
TSH with HGS	Standardized β	-0.039	0.055	-0.042
	Standard Error	0.374	0.447	0.511
	*t* Value	-1.447	1.450	-0.811
	*p* Value	0.149	0.148	0.418
		Model 3		
FT3/FT4 with HGS	Standardized β	0.005	0.091	0.087
	Standard Error	4.509	5.757	6.658
	*t* Value	0.200	2.428	1.697
	*p* Value	0.842	**0.016**	0.091
TSH with HGS	Standardized β	-0.046	0.035	-0.018
	Standard Error	0.374	0.440	0.523
	*t* Value	-1.730	0.945	-0.330
	*p* Value	0.084	0.345	0.741

Model 1: unadjusted; Model 2: sex, race/ethnicity, educational level, marital status were adjusted; Model 3: sex, race/ethnicity, educational level, marital status, smoking status, drinking status, hypertension, BMI, UIC were adjusted; Bolded values were statistically significant (P<0.05).

The subgroup analysis was conducted to further evaluate the relationship between related thyroid function and HGS based on sex. After adjusting for all covariates except for sex, subgroup analysis by sex (**Table 3**) revealed that the positive association between FT3/FT4 and HGS was observed in middle age group for both male and female participants (β=0.163, t=2.121, P=0.035; β=0.157, t=2.180, P=0.031). For female participants in old age group, there was a negative association between TSH and HGS in Model 1 (**Supplementary Table S1**). However, this negative association was not significant in Model 2 and Model 3. After stratifying sex, we, surprisingly, noticed that neither FT3/FT4 nor TSH was associated with HGS in young age group (all P>0.05).

**Table 3:** Subgroup analysis of the association between thyroid function and HGS among different age groups stratified by sex.

		Young age	Middle age	Old age
		Male		
		Model 3		
FT3/FT4 with HGS	Standardized β	-0.025	0.163	0.100
	Standard Error	7.251	9.213	10.983
	*t* Value	-0.453	2.121	0.978
	*p* Value	0.651	**0.035**	0.330
TSH with HGS	Standardized β	-0.085	0.100	0.062
	Standard Error	0.603	0.694	0.849
	*t* Value	-1.536	1.332	0.580
	*p* Value	0.126	0.185	0.563
		Female		
		Model 3		
FT3/FT4 with HGS	Standardized β	0.075	0.157	0.088
	Standard Error	4.910	4.278	7.640
	*t* Value	1.245	2.180	0.871
	*p* Value	0.215	**0.031**	0.387
TSH with HGS	Standardized β	-0.030	-0.064	-0.117
	Standard Error	0.404	0.543	0.572
	*t* Value	-0.477	-0.818	-1.202
	*p* Value	0.634	0.415	0.233

Results of subgroup analysis were adjusted for race/ethnicity, educational level, marital status, smoking status, drinking status, hypertension, BMI, and UIC (sex was not adjusted in this model); Bolded values were statistically significant (P<0.05).

### 3.3 Analysis of the non-linear relationship between thyroid function and HGS

**Figure 2:**
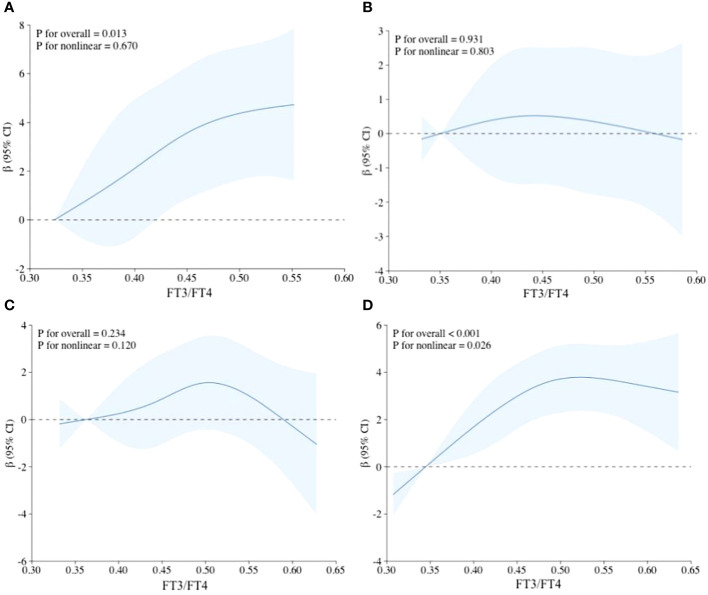
Non-linear relationship between FT3/FT4 and HGS using restricted cubic spline regression. **(A)** Adjusted RCS model in young age group; **(B)** Adjusted RCS model in middle age group; **(C)** Adjusted RCS model in old age group; **(D)** Adjusted RCS model in the overall population; Adjustments were made for sex, race/ethnicity, educational level, marital status, smoking status, drinking status, hypertension, BMI, and UIC.

In the restricted cubic spline regression (**Figure 2**), there was no significant non-linear association between FT3/FT4 and HGS in young, middle, and old age groups with all potential covariates fully adjusted. Interestingly, a statistically significant non-linear association was found between FT3/FT4 and HGS in overall population (P for non-linear=0.026). However, TSH had no significant non-linear relationship with HGS in both overall population and different age groups (**Supplementary Figure S1**). Although there were slightly different in results of the non-linear analysis, the overall tendency of β in each restricted cubic spline was relatively consistent.

### 3.4 Analysis of thyroid function in different age groups with or without low HGS stratified by sex

**Figure 3:**
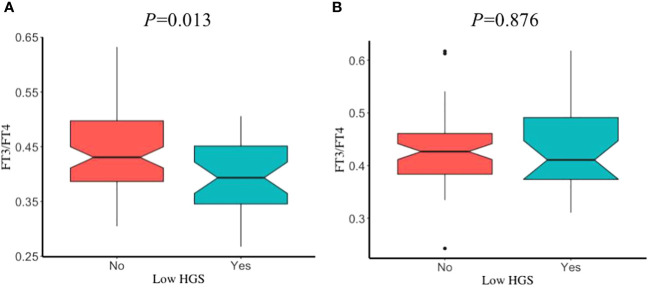
The comparisons in FT3/FT4 between participants with and without low HGS in old age group. **(A)** for euthyroid male; **(B)** for euthyroid female. Values were presented as the weighted mean with 95% CIs after adjusting race/ethnicity, educational level, marital status, smoking status, drinking status, hypertension, BMI, UIC.

**Figure 4:**
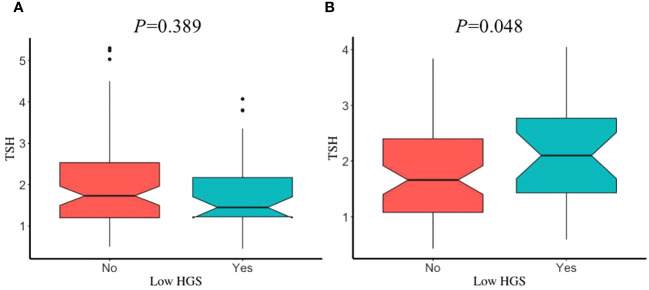
The comparisons in TSH between participants with and without low HGS in old age group. **(A)** for euthyroid male; **(B)** for euthyroid female. Values were presented as the weighted mean with 95% CIs after adjusting race/ethnicity, educational level, marital status, smoking status, drinking status, hypertension, BMI, UIC.

The prevalence of low HGS in young, middle, and old age groups was 39.51%, 38.07%, and 31.6%, respectively. Sex had no significant influence on participants with or without low HGS, in different age groups (all P>0.05). After adjustment for covariates, participants with low HGS had a significant lower FT3/FT4 than those without low HGS for males in old age group (P=0.013) (**Figure 3**), while this significant decrease was not observed for male in young and middle age groups (**Supplementary Figure S2**). However, FT3/FT4 was not significantly different between participants with and without low HGS for females among young, middle, and old age groups (all P>0.05). Interestingly, as shown in **Figure 4**, there was a significant increase in TSH level for female participants with low HGS in old age group compared to those with normal HGS (P=0.048). However, no statistically significant differences were found for TSH level between participants with or without low HGS in young and middle age groups (all P>0.05) (**Supplementary Figure S3**).

## 4 Discussion

To the best of our knowledge, it is the first cross-sectional study to explore the relationship between thyroid function and handgrip strength among euthyroid U.S. population using the NHANES database. A total of 1,067 participants were incorporated in our study, where a positive association among FT3/FT4 and HGS in middle age group was revealed as statistically significant, while the association between TSH and HGS was not observed significantly after adjustment for potential confounders. Interestingly, our results differed from previous findings. For example, the Mugello study conducted by Di Iorio et al. unveiled that FT3/FT4 within the lowest quartile (FT3/FT4<2.75) was inversely correlated with handgrip strength significantly in subjects aged more than 90 years (P=0.03) (22). Although the study design between these two studies was the same, participants in the former ranging from 45~65 years, and participants in the latter aged over 90 years. Furthermore, they divided FT3/FT4 into quartiles, and analysed the differences in the mean values of HGS between subjects in the FT3/FT4 quartiles of distribution, which differed from our study (FT3/FT4 was processed as continuous data and not split to quartiles). As such, the different findings between these two studies might be partly explained by the different investigated age groups and data analysis approaches between two studies. Additionally, A small-scale study of fifty-one fit adults demonstrated that FT3/FT4 ratio was negatively correlated with lower muscle strength (23). Because of the limited subjects, the evidence of this study is ranked low, and the findings should be considered cautiously. Importantly, Gu et al. found that a significant association was observed between FT3/FT4 ratio and annual HGS change in Chinese population aged older than 40 years (β=0.054, P=0.02) (13), which was consistent with our study. Taken together, Future longitudinal study should be conducted to validate the therapeutic function of FT3/FT4 to slow the loss of muscle strength and maintain physical well-being in U.S. population within the middle age due to the limitation of cross-sectional design in our study.

The sex-stratified subgroup analysis indicated that the association between FT3/FT4 and HGS was solely significant for men rather than women in middle age group. It is commonly recognized that androgens and estrogens contribute to sex-related discrepancy in skeletal muscle morphology and function (24). In addition, age-associated declines in muscle mass, muscle strength, and physical function and increases in fat mass are shown to be evidently occurred in men than in women (25, 26), suggesting that the muscle strength is more affected by thyroid function in men to some extent. However, the significant relationship was not found for TSH with HGS in either men or women of different age groups, which was inconsistent with findings of Kim et al. where TSH was positively associated with HGS in men aged ≥50 years (P = 0.032) (27), and Spira et al. where subjects with high TSH level had a significantly lower handgrip strength than those with TSH within the reference range (28). The difficulty of finding an association between TSH and HGS in our study might be explicated in part by the fact that the TSH is modulated by a variety of factors. TSH is mediated by TSH receptors (TSHR) whose expression can be also detected outside the thyroid including the cells of the immune system following infections or other factors (29). Thus, the association between TSH and muscle strength remains unclear due to its complexity of TSH function.

The physiological mechanisms pertaining to the relationship between thyroid function and muscle strength are relatively intricate and multifactorial in nature (30–32). Thus, we attempted to analyse the non-linear relationship between thyroid function and muscle strength using restricted cubic spline regression analysis to further decipher its association. A particular interesting result in RCS analysis was that a statistically significant non-linear association was observed between FT3/FT4 ratio and HGS in overall population, while this non-linear association was no longer significantly present after grouping participants to young, middle and old age tertiles. It is well established that skeletal muscle is one of the primary target organs of thyroid hormones (33). One critical role of T3 is to change the skeletal fiber from slow-twitch (type 1) to fast-twitch type (type IIa, IIx, IIb) by modulating the transcription activity of genes MYH1, 2, 4 and 7 (34, 35). Nevertheless, excessive thyroid hormones can repress the secretion of pituitary growth hormone and reduce the levels of IGF-1 (Insulin-like growth factor-1), which contributes to influencing muscle mass and function as reflected by mediating muscle growth and regeneration (36).

In order to further clarify the thyroid function under different conditions of muscle strength, we classified participants into normal HGS and low HGS using the formula proposed by Wang et al. Our results showed that the FT3/FT4 difference in participants with or without low HGS was only significantly observed in men in old age group. However, female participants with low HGS had a significant higher TSH level than those without low HGS in old age group. The findings of our study were inconsistent with Kim et al., where the difference of serum TSH level between subjects with and without low muscle strength was significant statistically in men (27). In their study, they expounded that this may be on account of the dramatic reduction in estrogens in women with the age and the sex difference to a large extent. Given the different findings between studies, future studies should be focused on exploring how sex-related factors regulate thyroid hormones associated with muscle function.

The strengths of our study were that the data were collected from the NHANES database in the U.S. National Center for Health Statistics, ensuring the representativity and generalizability of our study. Furthermore, it is the first study to focus on the U.S. population to determine the relationship between thyroid function and muscle strength in different age groups. However, it had to be acknowledged that there were some limitations. First of all, we did not examine the causal relationship between thyroid function and HGS due to the cross-sectional study design of the NHANES dataset. Future longitudinal cohort study should be done to reveal its causal relationship. Second, many other potential factors except for confounders aforementioned in our study could influence the relationship between these two variables. In addition, we excluded participants less than 20 years due to the unpredictability of thyroid gland development and low compliance of HGS examination in children. As such, future children-oriented studies should be conducted.

## 5 Conclusion

In conclusion, this study demonstrated a significant positive association between FT3/FT4 and HGS in middle age group. Interestingly, the different association was observed in men in middle age group after adjusting potential confounding factors when participants were stratified by sex. However, no statistically significant association was found between TSH and HGS with all covariates fully adjusted. Future longitudinal cohort study should be conducted to reveal the causal relationship between thyroid function and muscle strength.

## Data availability statement

The original contributions presented in the study are included in the article/**Supplementary Material**. Further inquiries can be directed to the corresponding author.

## Ethics statement

The studies involving humans were approved by the National Center for Health Statistics Research Ethics Review Board. The studies were conducted in accordance with the local legislation and institutional requirements. The participants provided their written informed consent to participate in this study.

## Author contributions

ZW: Conceptualization, Formal analysis, Writing – original draft. PW: Data curation, Methodology, Writing – review & editing. JY: Data curation, Methodology, Writing – review & editing. YJ: Data curation, Methodology, Writing – review & editing. JW: Data curation, Methodology, Writing – review & editing. CL: Conceptualization, Supervision, Writing – review & editing.

## References

[1] Yen PM. Physiological and molecular basis of thyroid hormone action. Physiol Rev (2001) 81:1097–142. doi: 10.1152/physrev.2001.81.3.1097 11427693

[2] Oppenheimer JH Schwartz HL Surks MI. Propylthiouracil inhibits the conversion of L-thyroxine to L-triiodothyronine. An explanation of the antithyroxine effect of propylthiouracil and evidence supporting the concept that triiodothyronine is the active thyroid hormone. J Clin Invest (1972) 51:2493–7. doi: 10.1172/JCI107063 PMC292418 4639029

[3] Stabouli S Papakatsika S Kotsis V. Hypothyroidism and hypertension. Expert Rev Cardiovasc Ther (2010) 8:1559–65. doi: 10.1586/erc.10.141 21090931

[4] Dugbartey AT. Neurocognitive aspects of hypothyroidism. Arch Intern Med (1998) 158:1413–8. doi: 10.1001/archinte.158.13.1413 9665349

[5] Biondi B Klein I. Hypothyroidism as a risk factor for cardiovascular disease. Endocrine (2004) 24:1–13. doi: 10.1385/ENDO:24:1:001 15249698

[6] Reddy V Taha W Kundumadam S Khan M. Atrial fibrillation and hyperthyroidism: A literature review. Indian Heart J (2017) 69:545–50. doi: 10.1016/j.ihj.2017.07.004 PMC5560908 28822529

[7] Miao CQ Wang WY Zhao Y Lu DC Meng XL. Hyperthyroidism presenting with coronary vasospasm. J Coll Physicians Surg Pak (2022) 32:1492–4. doi: 10.29271/jcpsp.2022.11.1492 36377023

[8] Cooper R Kuh D Hardy R. Mortality Review Group; FALCon and HALCyon Study Teams. Objectively measured physical capability levels and mortality: systematic review and meta-analysis. BMJ (2010) 341:c4467. doi: 10.1136/bmj.c4467 20829298 PMC2938886

[9] Newman AB Kupelian V Visser M Simonsick EM Goodpaster BH Kritchevsky SB. Strength, but not muscle mass, is associated with mortality in the health, aging and body composition study cohort. J Gerontol A Biol Sci Med Sci (2006) 61:72–7. doi: 10.1093/gerona/61.1.72 16456196

[10] Bohannon RW. Muscle strength: clinical and prognostic value of hand-grip dynamometry. Curr Opin Clin Nutr Metab Care (2015) 18:465–70. doi: 10.1097/MCO.0000000000000202 26147527

[11] Casas F Pessemesse L Grandemange S Seyer P Baris O Gueguen N. Overexpression of the mitochondrial T3 receptor induces skeletal muscle atrophy during aging. PloS One (2009) 4:e5631. doi: 10.1371/journal.pone.0005631 19462004 PMC2680484

[12] Ahn SH Seo DH Cho Y Jung M Kim SH Hong S. Different relationships between thyrotropin and muscle strength according to sex and age in euthyroid koreans (The 6th korea national health and nutritional examination survey 2014-2015). Thyroid (2020) 30:1710–7. doi: 10.1089/thy.2019.0601 32438894

[13] Gu Y Meng G Wu H Zhang Q Liu L Bao X. Thyroid function as a predictor of handgrip strength among middle-aged and older euthyroid adults: the TCLSIH cohort study. J Am Med Dir Assoc (2019) 20:1236–41. doi: 10.1016/j.jamda.2019.06.014 31422066

[14] Chen S Huang W Zhou G Sun X Jin J Li Z. Association between sensitivity to thyroid hormone indices and bone mineral density in US males. Int J Endocrinol (2022) 2022:2205616. doi: 10.1155/2022/2205616 36340930 PMC9629943

[15] Wang YC Bohannon RW Li X Sindhu B Kapellusch J. Hand-grip strength: normative reference values and equations for individuals 18 to 85 years of age residing in the United States. J Orthop Sports Phys Ther (2018) 48:685–93. doi: 10.2519/jospt.2018.7851 29792107

[16] Zhao S Gao W Li J Sun M Fang J Tong L. Dietary inflammatory index and osteoporosis: the National Health and Nutrition Examination Survey, 2017-2018. Endocrine (2022) 78:587–96. doi: 10.1007/s12020-022-03178-6 36044108

[17] Chobanian AV Bakris GL Black HR Cushman WC Green LA Izzo JL Jr. The Seventh Report of the Joint National Committee on Prevention, Detection, Evaluation, and Treatment of High Blood Pressure: the JNC 7 report [published correction appears in JAMA. JAMA (2003) 289:2560–72. doi: 10.1001/jama.289.19.2560 12748199

[18] Lang X Li Y Zhang D Zhang Y Wu N Zhang Y. FT3/FT4 ratio is correlated with all-cause mortality, cardiovascular mortality, and cardiovascular disease risk: NHANES 2007-2012. Front Endocrinol (Lausanne) (2022) 13:964822. doi: 10.3389/fendo.2022.964822 36060933 PMC9433660

[19] Liu N Ma F Feng Y Ma X. The association between the dietary inflammatory index and thyroid function in U.S. Adult males. Nutrients (2021) 13:3330. doi: 10.3390/nu13103330 34684331 PMC8540204

[20] Centers for Disease, Control, and Prevention. National Health and Nutrition Examination Survey. Survey Methods and Analytic Guidelines. Available online at: https://wwwn.cdc.gov/nchs/nhanes/analyticguidelines.aspx.

[21] Chung SM Moon JS Yoon JS Won KC Lee HW. Sex-specific effects of blood cadmium on thyroid hormones and thyroid function status: Korean nationwide cross-sectional study. J Trace Elem Med Biol (2019) 53:55–61. doi: 10.1016/j.jtemb.2019.02.003 30910207

[22] Di Iorio A Paganelli R Abate M Barassi G Ireland A Macchi C. Thyroid hormone signaling is associated with physical performance, muscle mass, and strength in a cohort of oldest-old: results from the Mugello study. Geroscience (2021) 43:1053–64. doi: 10.1007/s11357-020-00302-0 PMC8110652 33219914

[23] Emerenziani GP Izzo G Vaccaro MG Quattrone A Lenzi A Aversa A. Gender difference and correlation between sexuality, thyroid hormones, cognitive, and physical functions in elderly fit. J Endocrinol Invest (2019) 42:699–707. doi: 10.1007/s40618-018-0974-1 30406377

[24] Anderson LJ Liu H Garcia JM. Sex differences in muscle wasting. Adv Exp Med Biol (2017) 1043:153–97. doi: 10.1007/978-3-319-70178-3_9 29224095

[25] Janssen I Heymsfield SB Wang ZM Ross R. Skeletal muscle mass and distribution in 468 men and women aged 18-88 yr. J Appl Physiol (1985). (2000) 89:81–8. doi: 10.1152/jappl.2000.89.1.81 10904038

[26] Churchward-Venne TA Breen L Phillips SM. Alterations in human muscle protein metabolism with aging: Protein and exercise as countermeasures to offset sarcopenia. Biofactors (2014) 40:199–205. doi: 10.1002/biof.1138 24105883

[27] Kim BJ Lee SH Isales CM Hamrick MW Kwak MK Koh JM. Association of serum TSH with handgrip strength in community-dwelling euthyroid elderly. J Clin Endocrinol Metab (2018) 103:3986–92. doi: 10.1210/jc.2018-01095 30137405

[28] Spira D Buchmann N Demuth I Steinhagen-Thiessen E Völzke H Ittermann T. Association of thyroid function with handgrip strength: data from the study of health in pomerania and the berlin aging study II. Thyroid (2019) 29:1220–6. doi: 10.1089/thy.2018.0646 31426720

[29] Nagayama Y Takeshita A Luo W Ashizawa K Yokoyama N Nagataki S. High affinity binding of thyrotropin (TSH) and thyroid-stimulating autoantibody for the TSH receptor extracellular domain. Thyroid (1994) 4:155–9. doi: 10.1089/thy.1994.4.155 7919997

[30] Gruener R Stern LZ Payne C Hannapel L. Hyperthyroid myopathy. Intracellular electrophysiological measurements in biopsied human intercostal muscle. J Neurol Sci (1975) 24:339–49. doi: 10.1016/0022-510x(75)90254-3 1117309

[31] Brennan MD Powell C Kaufman KR Sun PC Bahn RS Nair KS. The impact of overt and subclinical hyperthyroidism on skeletal muscle. Thyroid (2006) 16:375–80. doi: 10.1089/thy.2006.16.375 16646684

[32] Wang K Zhang D Cao G Wang C Wang L Zhao R. A low free T3 to free T4 ratio is associated with sarcopenia in euthyroid patients with type 2 diabetes mellitus. J Diabetes Res (2022) 2022:2305156. doi: 10.1155/2022/2305156 36034587 PMC9402295

[33] Ambrosio R De Stefano MA Di Girolamo D Salvatore D. Thyroid hormone signaling and deiodinase actions in muscle stem/progenitor cells. Mol Cell Endocrinol (2017) 459:79–83. doi: 10.1016/j.mce.2017.06.014 28630021

[34] Simonides WS van Hardeveld C. Thyroid hormone as a determinant of metabolic and contractile phenotype of skeletal muscle. Thyroid (2008) 18:205–16. doi: 10.1089/thy.2007.0256 18279021

[35] Bloise FF Cordeiro A Ortiga-Carvalho TM. Role of thyroid hormone in skeletal muscle physiology. J Endocrinol (2018) 236:R57–68. doi: 10.1530/JOE-16-0611 29051191

[36] Giovannini S Marzetti E Borst SE Leeuwenburgh C. Modulation of GH/IGF-1 axis: potential strategies to counteract sarcopenia in older adults. Mech Ageing Dev (2008) 129:593–601. doi: 10.1016/j.mad.2008.08.001 18762207 PMC5992490

