# Supplementary material for: Serum FT3/FT4, but not TSH is associated with handgrip strength in euthyroid U.S. population: evidence from NHANES
Source: Front Endocrinol (Lausanne). 2024 Mar 4;15:1323026. doi: 10.3389/fendo.2024.1323026 (PMC10947195; doi:10.3389/fendo.2024.1323026)
Supplement: Supplementary file 1 [file DataSheet_1.zip › Supplementary figure S1.pdf]

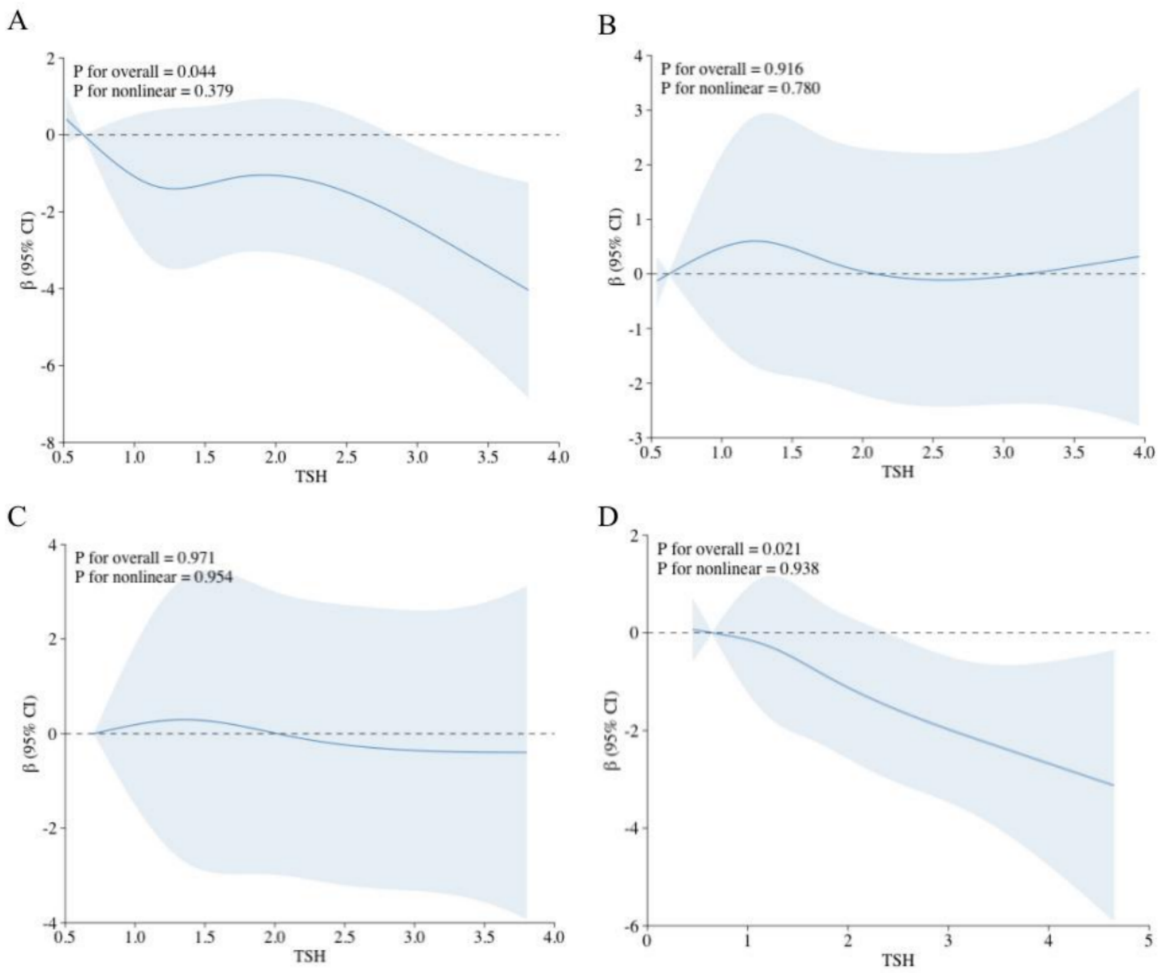

Figure S1. Non-linear relationship between TSH and HGS using restricted cubic spline regression. A: Adjusted RCS model in the young age group; B: Adjusted RCS model in the middle age group; C: Adjusted RCS model in the old age group; D: Adjusted RCS model in the overall population; Adjustments were made for sex, race/ethnicity, educational level, marital status, smoking status, drinking status, hypertension, BMI, and UIC.
