# Supplementary material for: Serum FT3/FT4, but not TSH is associated with handgrip strength in euthyroid U.S. population: evidence from NHANES
Source: Front Endocrinol (Lausanne). 2024 Mar 4;15:1323026. doi: 10.3389/fendo.2024.1323026 (PMC10947195; doi:10.3389/fendo.2024.1323026)
Supplement: Supplementary file 1 [file DataSheet_1.zip › Supplementary figure S3.pdf]

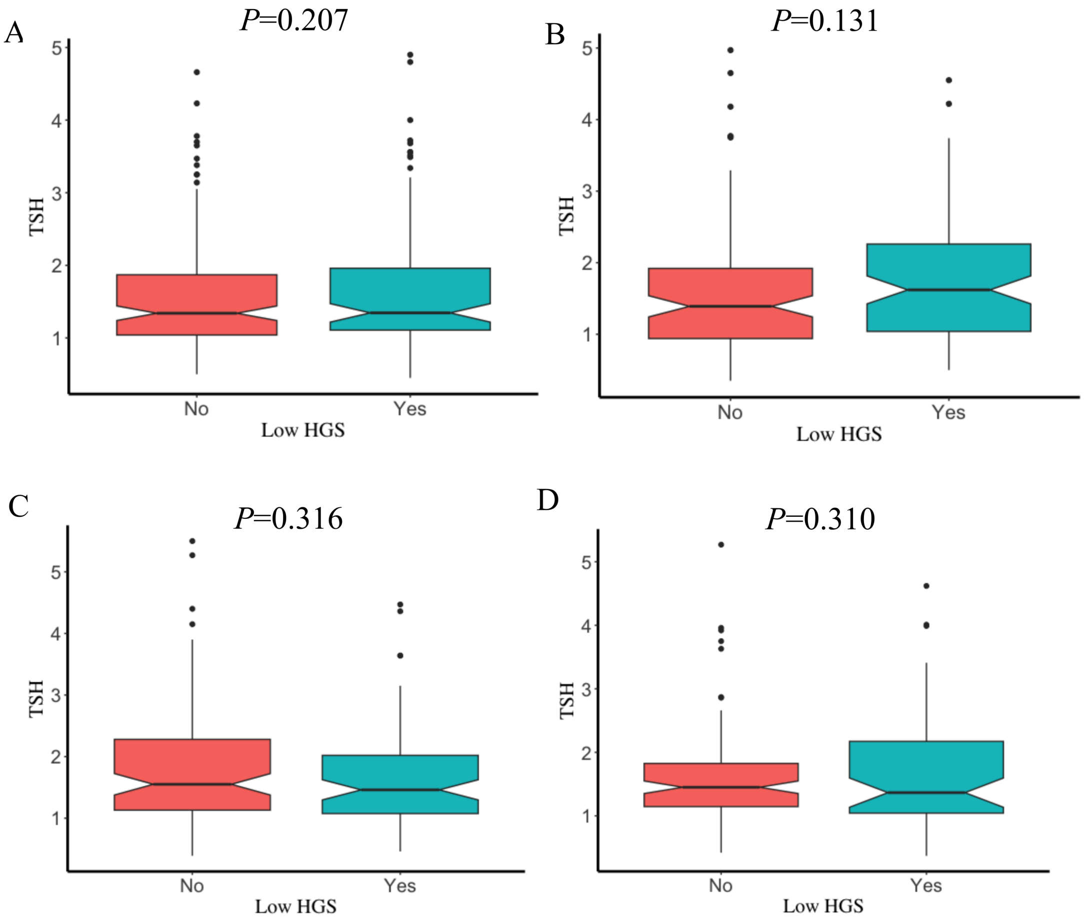


Figure S3. The comparisons in TSH between participants with and without low HGS in young and middle age groups. A: for euthyroid male in young age group; B: for euthyroid female in young age group; C: for euthyroid male in middle age group; D: for euthyroid female in middle age group. Values were presented as the weighted mean with 95% CIs after adjusting race/ethnicity, educational level, marital status, smoking status, drinking status, hypertension, BMI, UIC.
