# Supplementary material for: Serum FT3/FT4, but not TSH is associated with handgrip strength in euthyroid U.S. population: evidence from NHANES
Source: Front Endocrinol (Lausanne). 2024 Mar 4;15:1323026. doi: 10.3389/fendo.2024.1323026 (PMC10947195; doi:10.3389/fendo.2024.1323026)
Supplement: Supplementary file 1 [file DataSheet_1.zip › Supplementary table S1.pdf]

Table S1. Subgroup analysis of the association between thyroid function and HGS among different age groups stratified by sex in Model 1 and 2

		Young age	Middle age	Old age
			Male	
			Model 1	
FT3/FT4 with HGS	Standardized β	-0.0003	0.128	0.106
	Standard Error	7.228	8.789	10.240
	t Value	-0.005	1.750	1.117
	p Value	0.996	0.082	0.266
TSH with HGS	Standardized β	-0.096	0.141	-0.004
	Standard Error	0.598	0.695	0.757
	t Value	-1.743	1.878	-0.041
	p Value	0.082	0.062	0.967
			Model 2	
FT3/FT4 with HGS	Standardized β	-0.031	0.165	0.099
	Standard Error	7.042	9.392	10.765
	t Value	-0.571	2.105	0.994
	p Value	0.568	**0.037**	0.322
TSH with HGS	Standardized β	-0.078	0.156	0.013
	Standard Error	0.617	0.701	0.813
	t Value	-1.374	2.058	0.130
	p Value	0.171	**0.041**	0.897
			Female	
			Model 1	
FT3/FT4 with HGS	Standardized β	0.066	0.115	0.074
	Standard Error	5.072	6.695	7.939
	t Value	1.051	1.612	0.706
	p Value	0.295	0.109	0.482
TSH with HGS	Standardized β	-0.024	-0.063	-0.201
	Standard Error	0.433	0.523	0.553
	t Value	-0.347	-0.832	-2.139
	p Value	0.729	0.406	**0.035**
			Model 2	
FT3/FT4 with HGS	Standardized β	0.091	0.110	0.105
	Standard Error	4.851	6.695	7.829
	t Value	1.517	1.533	1.017
	p Value	0.131	0.127	0.312
TSH with HGS	Standardized β	-0.026	-0.083	-0.177
	Standard Error	0.416	0.528	0.581
	t Value	-0.398	-1.093	-1.792
	p Value	0.691	0.276	0.077

Results of subgroup analysis were adjusted for race/ethnicity, educational level, marital status, smoking status, drinking status, hypertension, BMI, and UIC (sex was not adjusted in this model); Bolded values were statistically significant ($P<0.05$).
